# In Vivo Antimicrobial and Wound-Healing Activity of Resveratrol, Dihydroquercetin, and Dihydromyricetin against *Staphylococcus aureus, Pseudomonas aeruginosa*, and *Candida albicans*

**DOI:** 10.3390/pathogens9040296

**Published:** 2020-04-17

**Authors:** Alexei B. Shevelev, Nicola La Porta, Elena P. Isakova, Stefan Martens, Yulia K. Biryukova, Alexander S. Belous, Dmitrii A. Sivokhin, Elena V. Trubnikova, Marina V. Zylkova, Alla V. Belyakova, Maria S. Smirnova, Yulia I. Deryabina

**Affiliations:** 1 Vavilov Institute of General Genetics, Russian Academy of Sciences, 117971 Moscow, Russia; shevel_a@hotmail.com (A.B.S.); dr.sivokhin@gmail.com (D.A.S.); tr_e@list.ru (E.V.T.); mary.zyl@mail.ru (M.V.Z.); allusya_uzmu@mail.ru (A.V.B.); mbarbotko@yandex.ru (M.S.S.); 2 IASMA Research and Innovation Centre, Fondazione Edmund Mach, 38010 San Michele all’ Adige, Italy; nicola.laporta@fmach.it (N.L.P.); stefan.martens@fmach.it (S.M.); 3 EFI Project Centre on Mountain Forests (MOUNTFOR), 38010 San Michele all’Adige, Italy; 4 Bach Institute of Biochemistry, Research Center of Biotechnology of the Russian Academy of Sciences, 119071 Moscow, Russia; elen_iss@mail.ru (E.P.I.); yul_der@mail.ru (Y.I.D.); 5 Scientific Research Laboratory “Genetics”, Kursk State University, 305000 Kursk, Russia; a.s.belous@mail.ru; 6 International School “Medicine of Future” of Biomedical Park of I. M. Sechenov, First Moscow State Medical University (Sechenov University), 119991 Moscow, Russia

**Keywords:** Resveratrol, Dihydroquercetin, Dihydromyricetin, polyphenols, antimicrobial activity, wound-healing activity

## Abstract

An increase in the spread of antibiotic-resistant opportunistic microorganisms causes serious problems in the treatment of purulent infections, burns, and trophic ulcers. We tested the antimicrobial activity in vivo of three polyphenols, Resveratrol, Dihydroquercetin (Taxifolin), and Dihydromyricetin (Ampelopsin) from Norway spruce bark to promote the elimination of *Staphylococcus aureus, Pseudomonas aeruginosa,* and *Candida albicans* from wounds. Purulent infection was modelled on wounds in rats infected with suspensions containing 10^9^ CFU (colony-forming unit)/mL of pathogens. The wound area was treated daily with solutions of the polyphenols or placebo for 14 days after the beginning of the treatment. The animals were examined daily, and each stage of the wound healing (inflammation, granulation, and maturation (marginal epithelialisation) was documented. The planimetric analysis of the wound recovery percentage was performed on the 3rd, 10th, and 14th day after the start of curing. Then, one echelon (three or four animals from each subgroup) was withdrawn from the experiment on days 3 (three animals), 10 (three animals), and 14 (four animals) for microscopy analysis of cytological composition of their wound defects by microscopy and microbiological analysis of their contamination with pathogens. Our results show that they are also able to suppress mast cell infiltration and stimulate lymphocyte and macrophage (monocyte) infiltration into the wound. Resveratrol stimulated the replacement of the scar with normal tissue (with a clear boundary between the dermis and epidermis) and the restoration of hair follicles. Resveratrol turned out to be significantly better than some commercial antimicrobial (Levomecol) and antifungal (Clotrimazole) ointments and can be proposed as a promising drug for topical use for the treatment of trophic ulcers and burns.

## 1. Introduction

Due to the broad use of antibiotics, drug resistance of microbial pathogens has become one of the greatest problems of modern medicine. It is of particular importance under complicated chronic conditions such as in trophic ulcers in patients with diabetes mellitus where long-term antimicrobial therapy is mandatory [1]. Therefore, it is necessary to search for new antibacterial and antifungal agents that will be safe for the patient, effectively destroy the pathogen, and promote wound healing.

Plant-derived stilbenoids, especially Resveratrol and its derivatives, have become broadly known for their positive effects on a wide range of medical disorders reported in many published studies [2,3]. First, Resveratrol and Dihydroquercetin were isolated from grape juice and peanut and coniferous tree bark extracts [4] as agents responsible for the response to a fungus *Botrytis cinereae* infection in plants. Later, these compounds were supposed to be responsible for the so-called French paradox [5] and were included in pharmacopoeia. The reports about the isolation of polyphenols from coniferous tree bark and wood appear episodically [6].

The high activity of Resveratrol and its 13 analogues against grape pathogens growing on plants is shown [7]. Some authors have reported the activity of a synthetic resveratrol-trans-dihydrodimer antimicrobial against the Gram-positive bacteria of *Bacillus cereus*, *Listeria monocytogenes*, and *Staphylococcus aureus* (minimal inhibitory concentration (MIC)  =  15.0, 125, and 62.0 μM, respectively) and against Gram-negative *Escherichia coli* (MIC  =  123 μM, upon the addition of the efflux pump inhibitor of Phe-Arg-β-naphthylamide) [8]. They suggested that the antibacterial activity of Resveratrol should be realised due to:

(1) The disruption of membrane potential in bacteria.

(2) Blocking of DNA synthesis.

Chambers et al. proved the high activity of many plant polyphenols against *S. aureus* in vitro, including MRSA (Methicillin-resistant *S. aureus*) [9]. They concluded that many of the compounds inhibited the *S. aureus* multidrug resistance pump NorA and sensitised multidrug-resistant cancer cell lines showing the potential of Gallic acid as an adjuvant.

In a previous study, we showed the effectiveness of not only Resveratrol but also Dihydroquercetin and Dihydromyricetin against *S. aureus*. On the contrary, the effect on *Pseudomonas aeruginosa* and *C. albicans* was absent [10]. These data are in agreement with previous results [11], which showed the pronounced antimicrobial activity of Resveratrol against Gram-positive bacteria *B. cereus*, *Enterococcus faecalis,* and *S. aureus* and zero activity against the Gram-negative human pathogens *E. coli, Klebsiella pneumoniae, Salmonella typhimurium,* and *P. aeruginosa*.

In addition, Resveratrol is effective against *Campylobacter jejuni* [12], *B. cinerea* [13], herpes virus HSV-1 and HSV-2 [14], and others [15]. However, these authors do not report trials of the antibacterial efficacy of these compounds in vivo using animal models.

Taylor et al. tested the antimicrobial activity of Resveratrol against anaerobic species *Propionibacterium acnes* (phylum Actinobacteria), a causative agent of acne vulgaris, while benzoyl peroxide, a commonly used antibacterial medicine for acne, demonstrated a short-term bactericidal response [16]. The combination of Resveratrol and benzoyl peroxide showed a high initial antibacterial activity and sustained bacterial growth inhibition. Some authors reported that the concentrations of Resveratrol and some other phenolic compounds (kaempferol, quercetin, taxifolin, other stilbenes, and picein) in *Picea abies* (Norway spruce) change upon rust infection with *Chrysomyxa rhododendri* (class Pucciniomycetes) and increase plant resistance to phytopathogens [17].

Li et al. showed the substantial antifungal activity of Gallic acid, a major polyphenolic compound found in the peel of pomegranate (*Punica granatum)*, where the intraperitoneal injection of Gallic acid (80 mg/kg) significantly enhanced the cure rate in mice infected with *Candida albicans* [18].

Dihydromyricetin and its derivatives could only be isolated from coniferous wood and bark. They are less thoroughly investigated than Resveratrol and Dihydroquercetin; however, there are some studies where a similar action of the three polyphenols on *S. aureus* is shown [19].

According to our information, published data about the external application in vivo of Resveratrol, Dihydroquercetin, and Dihydromyricetin as antimicrobial drugs for animals are not available.

Taking into account some evident contradictions between numerous reports about the high antimicrobial activity of Resveratrol, Dihydroquercetin, and Dihydromyricetin in vitro and the lack of respective confirmations of its activity in vivo, we chose biological trials of these polyphenols using the models of Gram-positive (*S. aureus*), Gram-negative (*P. aeruginosa*), and fungal (*C. albicans*) pathogens as an objective for the present study.

## 2. Results

### 2.1. Wound-Healing Effects of Resveratrol, Dihydroquercetin, and Dihydromyricetin in Sterile Wounds

The data for the visual examination and planimetric assay of the compounds studied using sterile wounds (Table 1, Table 2 and Table 3) showed an evident acceleration of uninfected wound healing following treatment with Resveratrol, which is unachievable for Dihydroquercetin and Dihydromyricetin during the early stages of treatment. At the end of the experiment, the recovery rate in the Resveratrol subgroup decreased compared to other subgroups, perhaps due to the much smaller residual square of the wound at the moment, which limited the absolute rate of recovery.

**Figure 1:**
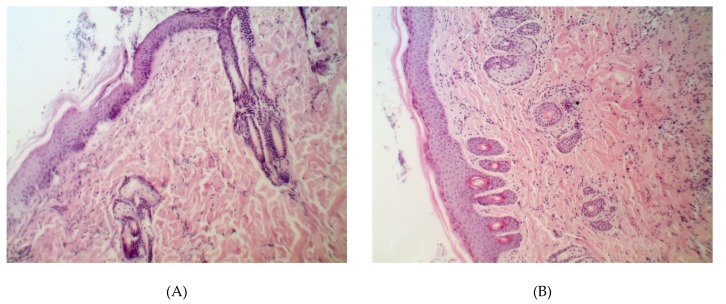

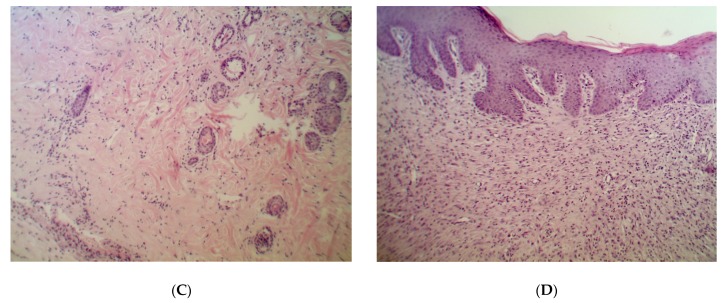
Histological structure of partially repaired damage in the animals with uninfected wound on the 14th day after the beginning of the treatment. Groups: (**A**) placebo; (**B**) Resveratrol; (**C**) Dihydroquercetin; (**D**) Dihydromyricetin. Magnification 200×.

The evidence from histological analysis confirmed the best wound-recovering ability of Resveratrol vs. the placebo, Dihydroquercetin, and Dihydromyricetin on the 14th day after the beginning of the treatment (Figure 1).

The treatment with Resveratrol restored the normal stratification of the dermis, epidermis, and hair bulbs, whereas a mature scar was observed in the other groups.

**Figure 2:**
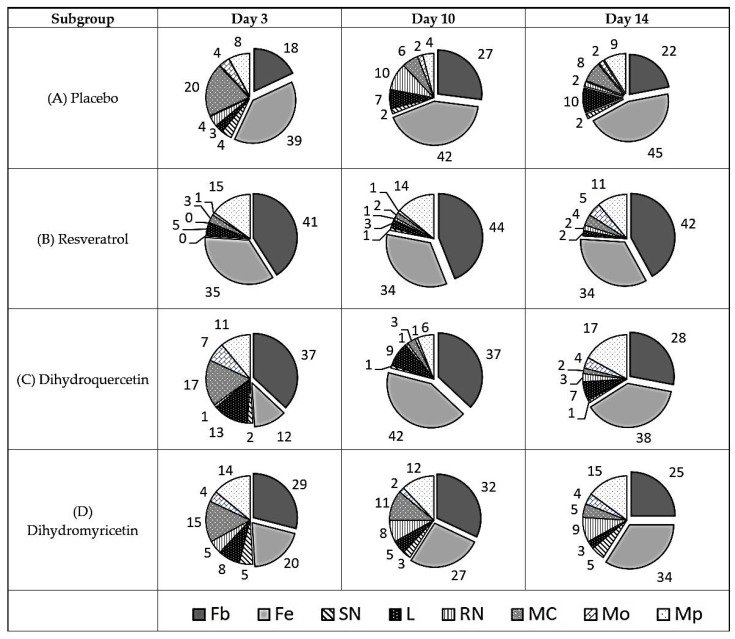
Diagram of cell types share in the cell repertoire of the uninfected wound defect surface, %. Cell types are denoted as follows: Fb—fibroblast; Fc—fibrocyte; SN—polymorphonuclear neutrophil; L—lymphocyte; RN—immature neutrophil; MC—mast cell; Mo—monocyte; Mp—macrophage. Total share of resident cells (Fb + Fc) is shown in box beneath respective sectors.

Since direct microbiological assays of the antibacterial impact in vivo using the method of bacteriological seeding resulted in poor interpretable data due to large variation between the animals studied, and probably due to the non-uniform distribution of bacterial growth focusing around the wound surface, an indirect method of assessment was used. The diagram of cell type share in the cell repertoire of the defective surface of the uninfected wound is shown in Figure 2.

The results in Figure 2 show that the recovery of the uninfected wound on day three was accompanied by consistent elevation of the fibroblast line from 59–69%, short-term elevation in the mast cell share on the 3rd day with a subsequent decrease in the share to the normal number by day 14, and the persistent growth of lymphocytes from 3% on the 3rd day up to 10% on day 14. The share of macrophages varied from 8–9%.

The treatment of uninfected wounds with Resveratrol provides quicker normalisation of the cell repertoire in the wound cavity. The share of neutrophils, lymphocytes, and mast cells in particular almost corresponds to that of unaffected skin. 

However, the share of macrophages grows greatly, as does the share of fibroblasts and fibrocytes. This is a good illustration of pathways providing the wound healing activity of Resveratrol.

On the contrary, Dihydroquercetin does not suppress the infiltration of mast cells, lymphocytes, and neutrophils and poorly stimulates activation of the transformation of monocytes into tissue macrophages.

Dihydromyricetin had similar effects to Resveratrol in uninfected wounds, but the degree of effect was lower.

An evident advantage of Resveratrol as a candidate in wound-healing medicine in comparison to Dihydromyricetin and Dihydroquercetin is confirmed by the overall share of resident cells (fibroblasts and fibrocytes) in the defect area on the 14th day of the experiment. It comprises 76% in the Resveratrol subgroup versus 67% in the placebo, 64% in the Dihydroquercetin, and 59% in the Dihydromyricetin groups.

### 2.2. Antimicrobial Effect of Resveratrol, Dihydroquercetin, and Dihydromyricetin Against S. aureus

**Figure 3:**
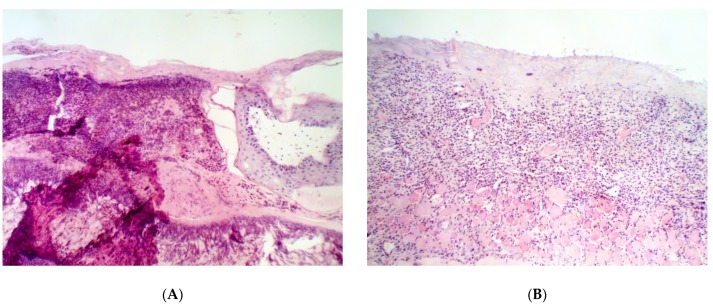

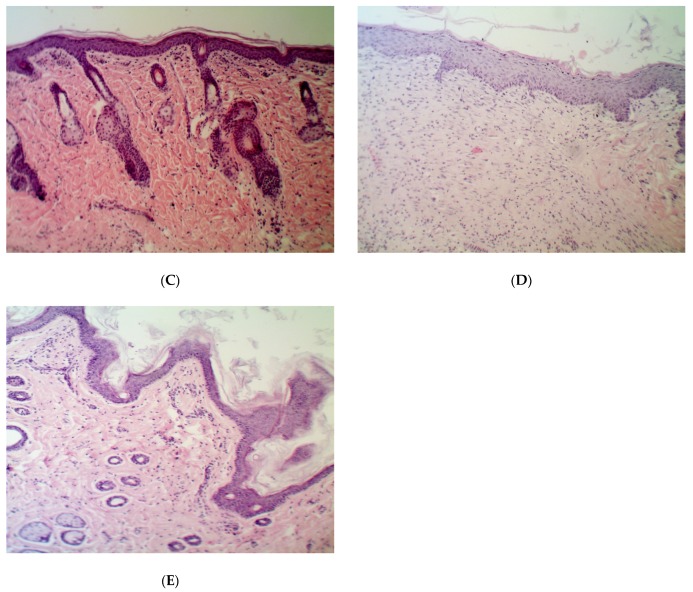
Histological structure of the partially repaired wound defect in the animals infected with *S. aureus* on the 14th day after the beginning of the treatment. Groups: (**A**) placebo; (**B**) control; (**C**) Resveratrol; (**D**) Dihydroquercetin; (**E**) Dihydromyricetin. Magnification 200×.

The rate of wound healing from the 1st to the 10th day of curing in all experimental subgroups was higher than in the placebo and control subgroups (Table 4, Table 5 and Table 6). However, the healing rate decreased at the end of the experiments (days 10–14) in the control subgroup and the Dihydroquercetin and Dihydromyricetin experimental groups. In the Resveratrol subgroup, it increased on days 10–14. Histological analysis on days 3 and 10 did not reveal a reliable difference between the control and experimental groups (data not shown), although the elimination of inflammation was much faster in the latter groups than in the placebo group. The analysis on day 14 (Figure 3) indicated a more evident defect recovery in the Resveratrol group (Figure 3C), which was the only group in which hair bulbs became distinguishable. Contrary to the control group, the border between the epidermis and dermis was clearly visible in those groups (Figure 3D,E). In the placebo group, only some traits of inflammation (neutrophil and lymphocyte infiltration, fibrin clots) were observed by the 14th day.

The data in Table 7 indicate that *S. aureus* 592 loads in the experimental subgroups of Resveratrol and Dihydroquercetin are lower than in the other three groups (placebo, control, and Dihydromyricetin). However, the conclusion has no statistical confidence due to the large variation between animals in all of the groups. Surprisingly, the use of a reference antibiotic (Levomecol) did not provide a statistically significant effect on the bacterial load in the wounds compared to placebo for the same reason.

**Figure 4:**
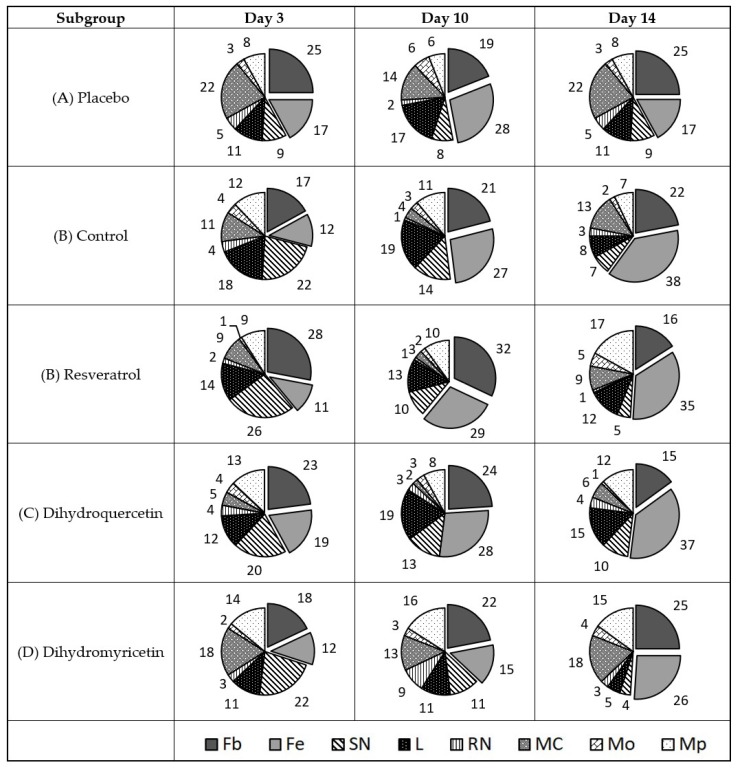
Diagram of cell types share in the cell repertoire of the defect surface in wounds infected with *Staphylococcus aureus*, %. Cell types are denoted as follows: Fb—fibroblast; Fc—fibrocyte; SN—segment-nuclear neutrophil; L—lymphocyte; RN—rod-nuclear neutrophil; MC—mast cell; Mo—monocyte; Mp—macrophage.

The data in Figure 4 illustrate a mechanism of antimicrobial action in vivo. The comparison of uninfected wounds (Figure 2) and those infected with *S. aureus* (Figure 4) allows us to conclude that infection causes a steady rise in the mast cell share, whereas the effect is observed on the 3rd day in sterile wounds. Conversely, the share of lymphocytes persistently rose in uninfected wounds but was decreased in those infected with *S. aureus*. This observation may be related to the well-known immune-depressant action of *S. aureus.* Moreover, the share of polymorphonuclear and immature neutrophils is stably low in the *S. aureus*-infected wounds, although *S. aureus* is a typical agent of purulent infections. The share of monocytes and macrophages is stable and low in both cases.

The treatment of wounds infected with *S. aureus* leads to substantial rearrangements in the cell repertoire in the wound. The treatment with Levomecol results in a drastic increase in the share of polymorphonuclear neutrophils, lymphocytes, and macrophages during the early curing stages. By day 14, the share of neutrophils, lymphocytes, and macrophages decreases. The share of mast cells is consistently much lower than that in the placebo-treated wounds infected with *S. aureus.* Taken together, the observations indicate a favourable direct antibacterial action (fewer mast cells, increase/decrease in the share of polymorphonuclear neutrophils) combined with an unfavourable immune depressant action (decrease in the share of lymphocytes and macrophages).

The assay of antimicrobial and immune modifying effect of the compounds studied in the wounds infected with *S. aureus* is shown in Figure 4.

The treatment of wounds infected with *S. aureus* with Resveratrol causes a drastic rise and then decrease in polymorphonuclear neutrophils and mast cells. However, the immune stimulation effect is stronger than the immune suppressant one since the share of lymphocytes is stable during the entire experiment and the share of macrophages persistently increases from the 3rd to the 14th day. The latter factor may contribute to the observed rapid elimination of fibrin clots and fine remodelling of the collagen matrix.

Dihydroquercetin has a similar impact to Resveratrol on the cell repertoire in wounds infected with *S. aureus.* It also gradually suppresses the share of neutrophils and mast cells, whereas the share of lymphocytes and macrophages remains rather high and stable during the entire experiment.

Dihydromyricetin is the least efficient of the three polyphenols studied for the treatment of wounds infected with *S. aureus*. Its effect on macrophages is similar to that of the other polyphenols, but the share of mast cells decreases much less. The share of lymphocytes also decreases; however, the share of segment nuclear lymphocytes on the 14th day of the experiment is low and corresponds to normal levels. This allows for a hypothesis that Dihydromyricetin possesses a direct antimicrobial efficacy against *S. aureus* rather than an immune-stimulating activity inherent to both Resveratrol and Dihydroquercetin.

### 2.3. Antimicrobial Effect of Resveratrol, Dihydroquercetin, and Dihydromyricetin Against P. aeruginosa

As for the group of animals infected with *S. aureus*, the rate of wound healing from the 1st to the 10th days of curing in all the experimental groups was higher than that in the placebo and control groups. The healing rate decreased at the end of the experiments (on the 10th–14th days) in the control groups, as well as in the Dihydroquercetin and Dihydromyricetin experimental groups, whereas it evidently increased in the Resveratrol group during this period (Table 8, Table 9 and Table 10).

**Figure 5:**
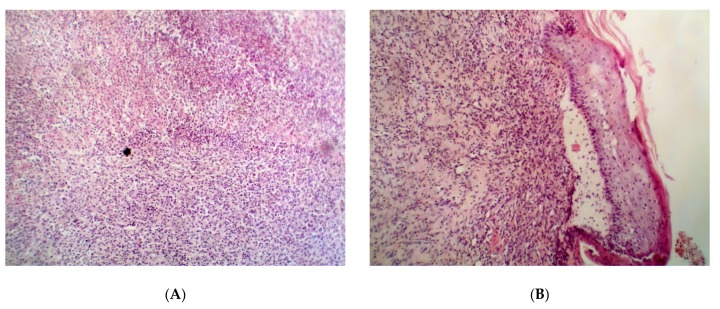

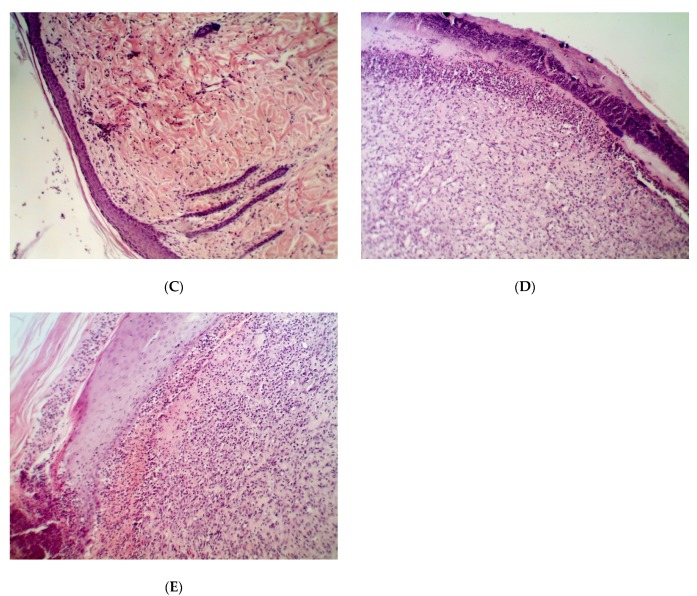
Histological structure of partially repaired wound defect in the animals infected with *P. aeruginosa* on the 14th day after the beginning of the treatment. Groups: (**A**) placebo; (**B**) control; (**C**) Resveratrol; (**D**) Dihydroquercetin; (**E**) Dihydromyricetin. Magnification 200×.

The results of clinical and planimetry assays are in good agreement with the analysis of histological sections (Figure 5). In contrast to wounds infected with *S. aureus*, the difference between the treated and untreated wounds infected with *P. aeruginosa* was rather weak. On the one hand, spontaneous recovery in the placebo group occurred faster, while on the other hand, even the treatment with Levomecol, Dihydroquercetin, and Dihydromyricetin did not result in the elimination of leukocytes and fibrin clot infiltration from the deep layers of the derma. It should be noted that the recovery of the epidermis in the *P. aeruginosa* group was faster and more complete than in the *S. aureus* group. The Resveratrol subgroup of the *P. aeruginosa* group had the lowest residual leukocyte infiltration, and this was the only group where the restoration of hair follicles on day 14 was detected (Figure 5C).

The results of microbiological assays of infected wounds indicate that *P. aeruginosa* (Table 11) load in the control, Resveratrol, and Dihydromyricetin subgroups is lower than that in the other subgroups (the placebo and Dihydroquercetin groups). It is likely that this conclusion in the *S. aureus* group has no statistical confidence due to a high individual average with all groups.

**Figure 6:**
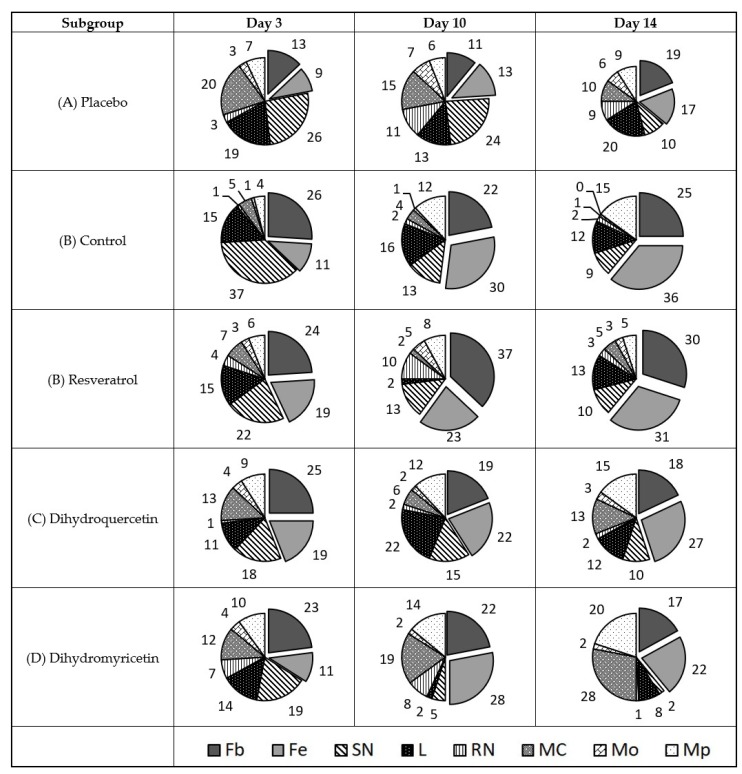
Diagram of cell types’ share in the cell repertoire of the wound defect surface is shown in the wounds infected with *P. aeruginosa*, %. Cell types are denoted as follows: Fb—fibroblast; Fc—fibrocyte; SN—polymorphonuclear neutrophil; L—lymphocyte; RN—immature neutrophil; MC—mast cell; Mo—monocyte; Mp—macrophage. Total share of resident cells (Fb + Fc) is shown in box beneath respective sectors.

The diagram of cell type share in the cell repertoire of the wound defect surface in wounds infected with *P. aeruginosa* is shown in Figure 6.

In contrast to *S. aureus*, the infection of wounds with *P. aeruginosa* leads to a less massive and constant infiltration of mast cells. In turn, the infiltration of neutrophils and lymphocytes in the wounds infected with *P. aeruginosa* is much higher (Figure 6). The infiltration of macrophages is rather weak. The Levomecol treatment of the wounds infected with *P. aeruginosa* almost normalises the share of the mast cells. The share of segment nuclear neutrophils in the presence of this therapeutic agent grows crucially on the 3rd day of the experiment and rapidly decreases by the 14th day. The share of macrophages and lymphocytes becomes steadily high. This immune-stimulating effect of the antibiotic could be explained by suppression of the innate immune-depressant functions of the bacteria once its viability is disturbed.

The efficacy of Resveratrol using such a model is confirmed by a strong effect on the suppression of nuclear neutrophil segments and mast cell infiltration. The share of lymphocytes is higher than that in the placebo group but lower than that in the control subgroup. The proportion of macrophages is also low.

Dihydroquercetin showed a moderately favourable impact on changes in the share of neutrophils, lymphocytes, and mast cells, whereas the stimulation of monocyte infiltration and their transformation into the macrophages using a model of *P. aeruginosa*-infected wounds was clearly shown.

The efficacy of Dihydromyricetin on the wounds infected with *P. aeruginosa* was accompanied by a rapid decrease in the share of segment nuclear neutrophils, whereas the share of mast cells increased and reached a supercritical value of 28% by the 14th day. The share of macrophages was also high. This defines Dihydromyricetin as a weak antimicrobial agent for the suppression of *P. aeruginosa;* however, it is a relatively active immunostimulant.

### 2.4. Antifungal Effect of Resveratrol, Dihydroquercetin, and Dihydromyricetin Against C. albicans

The data of planimetry and visual clinical assays of the compounds studied on *C. albicans* (Table 12, Table 13 and Table 14) showed that the infection of wounds with *C. albicans* leads to a much greater retardation in the healing of wounding defects than those with both *S. aureus* (Table 6) and *P. aeruginosa* (Table 10). Commercial antifungal ointment of Clotrimazole only moderately reduces recovery. In contrast, the recovery of infected wounds using the treatment with Resveratrol reached the level of recovery for uninfected wounds. The efficacy of both Dihydroquercetin and Dihydromyricetin was approximately the same as that in the control group; however, Dihydromyricetin provided a better wound-healing effect during the early stages, whereas Dihydroquercetin did this during the late stages of recovery. In this aspect, Dihydromyricetin is closer to Clotrimazole than to Dihydroquercetin.

**Figure 7:**
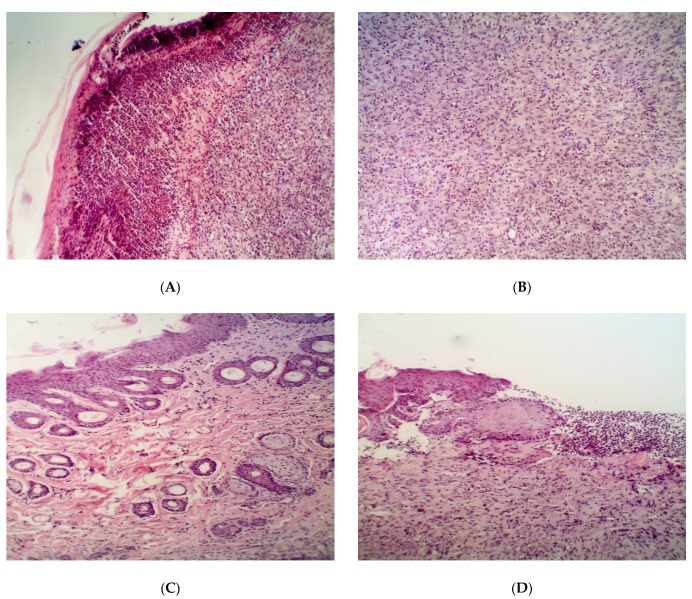

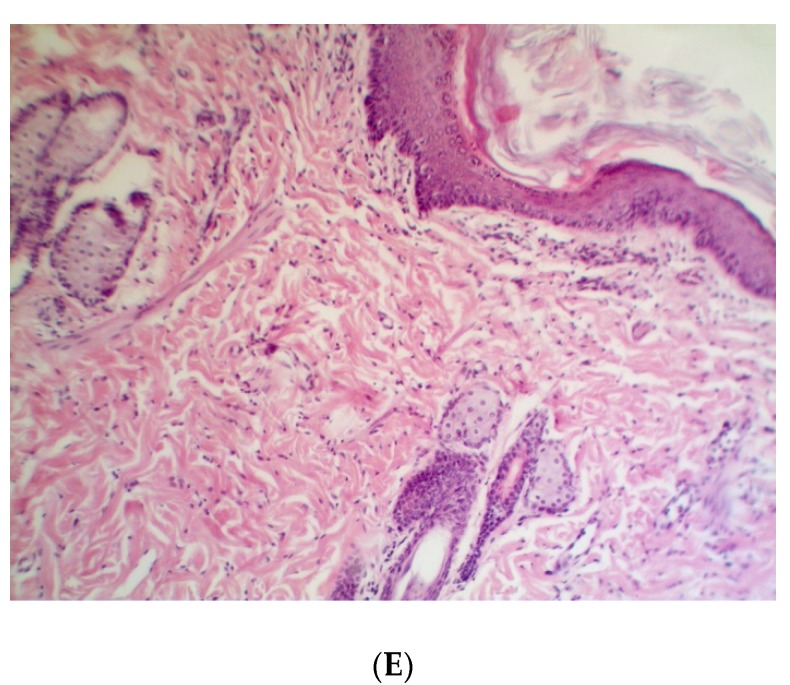
Histological structure of partially repaired wound defect in the animals infected with *C. albicans* on the 14th day after the beginning of the treatment. Groups: (**A**) placebo; (**B**) control; (**C**) Resveratrol; (**D**) Dihydroquercetin; (**E**) Dihydromyricetin. Magnification 200×.

Histological examination of the defects upon recovery showed that infection of the wounds with *C. albicans* did not stop spontaneously for 14 days. In Figure 7A, massive infiltration of the mast cells at the stage of degranulation is visible. Therefore, the infection with *C. albicans* looks more resistant and aggressive than that with bacterial pathogens. Treatment with the reference preparation provided no elimination of the inflammation by the 14th day of the experiment, and massive infiltration of the mast cells is observed. The results of microbiological assay (Table 15) show that, as in the case of bacterial infection, distribution of the *C. albicans* yeast was uneven, and a more accurate quantification of antibacterial efficacy of the drugs requires wider experimental groups. However, the growth of yeast load from the 3rd to 10th days and its subsequent return to an undetectable level by the 14th day is clearly seen in all subgroups. The presence of small or extensive inflammation that focuses in all of the subgroups, except for the Resveratrol and Dihydromyricetin ones (Figure 7), enables us to conclude that the complete elimination of *C. albicans* from the wounding defect by the 14th day is doubtful. However, a substantial reduction of the yeast cell load in the wound is evident. The best result was clearly obtained in the Resveratrol subgroup vs. the other subgroups (including the Control one).

**Figure 8:**
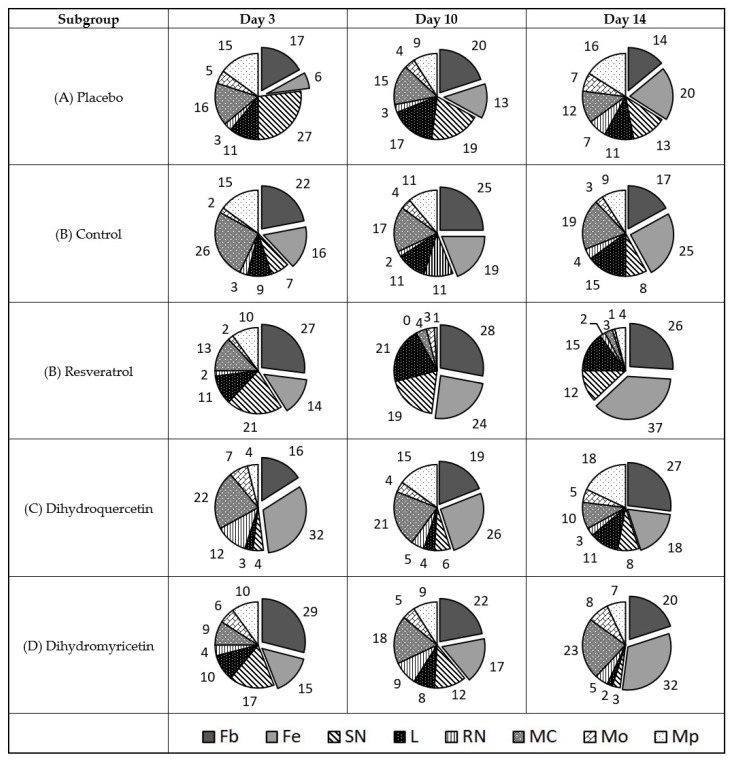
Diagram of cell types share in the cell repertoire of the defect surface in wounds infected with *C. albicans*, %. Cell types are denoted as follows: Fb—fibroblast; Fc—fibrocyte; SN—polymorphonuclear neutrophil; L—lymphocyte; RN—immature neutrophil; MC—mast cell; Mo—monocyte; Mp—macrophage. Total share of resident cells (Fb + Fc) is shown in box beneath respective sectors.

The antimicrobial assay and immune modifying effect of the compounds studied using the model of wounds infected with *C. albicans* is shown in Figure 8.

The data in Figure 8 partially explain the mechanism of action of the studied polyphenols against a fungal pathogen of *C. albicans.* Contrary to bacterial pathogens, infection of the wounds with *C. albicans* is accompanied by the massive infiltration of monocytes and their rapid transformation into macrophages. The infiltration of segment nuclear neutrophils and mast cells is observed, but it is less obvious than that in the case of bacterial infection. The infiltration of lymphocytes was steadily moderate. The level of fibroblasts and fibrocytes was much lower than that in the case of bacterial infection, which confirms more severe inflammation in the case of fungal infection.

Treatment with Clotrimazole reduced the infiltration of segment nuclear neutrophils and augmented the share of the mast cells and macrophages. The share of lymphocytes did not change compared to that in the placebo subgroup.

Unlike the reference drug, Resveratrol efficiently suppresses the infiltration of mast cells and macrophages, whereas the infiltration of nuclear segment neutrophils decreases moderately. The share of lymphocytes increased by the 10th day but remained unaffected by the 3rd and 14th days versus that in the placebo subgroup.

In contrast to Resveratrol, the treatment with Dihydroquercetin drastically reduced the infiltration of segment nuclear neutrophils, whereas the infiltration of mast cells, monocytes, and macrophages substantially increased compared to that in the placebo subgroup. The share of lymphocytes and immature neutrophils in this subgroup increased during the late and early stages of the treatment, respectively.

The treatment with Dihydromyricetin reduced the infiltration of all types of leukocytes to the wound area (hence, the share of fibroblasts and fibrocytes increased in this subgroup). The effect looks favourable since it means reducing the infection symptoms (pain and inflammation) immediately after the appearance of the injury. However, the progressive increase of the mast cell infiltration when day 10 is compared to day 3, and day 14 is compared with day 10, can lead to the hypothesis that such an immunosuppressive effect facilitates the formation of refugees for pathogenic agents and may delay an eventual recovery.

An unambiguous advantage of Resveratrol as a candidate antifungal medicine in comparison to Dihydromyricetin, Dihydroquercetin, and Clotrimazol is confirmed by the overall share of resident cells (fibroblasts and fibrocytes) in the defect area at day 14 of the experiment. It comprises 63% in the Resveratrol subgroup versus 34% in the placebo, 42% in the control, 45% in the Dihydroquercetin, and 52% in the Dihydromyricetin groups.

## 3. Discussion

To the best of our knowledge, the present study is the first to report the trials of antimicrobial efficacy of plant polyphenols in vivo as drugs for external use. First of all, the difference between the spectra of in vivo and in vitro antimicrobial activity appears rather impressive. We have previously reported that three polyphenols show high activity against a Gram-positive bacterium *S. aureus* in vitro, whereas the Gram-negative species *P. aeruginosa* and fungus *C. albicans* were not sensitive to them [10]. In this study, both pathogens were highly susceptible to the antimicrobial action of the polyphenols in vivo. Such an observation determines the need for in vivo trials, although they are more complicated and related to ethical concerns. The antifungal activity of Resveratrol against *B. cinerea* and some other fungal phytopathogens was shown upon its discovery. However, the present study seems to be the first to report the applicability of Resveratrol for the treatment of candidiasis in animals (and probably in humans).

The comparison of Resveratrol with the polyphenols of Dihydroquercetin and Dihydromyricetin showed its unambiguously higher efficacy using the three models of infected wounds (*S. aureus*, *P. aeruginosa*, *C. albicans*). It makes speculating about the applicability of Resveratrol as a prospective pharmaceutical substance for trophic ulceration and burn treatment possible. These injuries are often accompanied by combined bacterial and fungal infection and require the long-term administration of antibacterial medicines. Resveratrol meets at least several requirements as an antimicrobial for this purpose. It suppresses both fungal and bacterial pathogens, which is a rare trait for traditional antibiotics. Resveratrol is not expected to be sensitive to standard multiple drug resistance determinants since its molecular target is likely to be located on the outer cell surface and does not depend on efflux mechanisms [9]. Unlike most antibiotics, Resveratrol is not toxic for the patient, even when being administered for a long time [20]. Its effect is localised, so it should not affect mucosal microflora if being applied as a medicine for external use.

The obtained results showed that the studied polyphenols of Dihydroquercetin and Dihydromyricetin cannot compete with Resveratrol in the long-term treatment of infected wounds. However, both of them possess their own physiological activities and could be used as additives to Resveratrol. Dihydromyricetin manifested some immunostimulating and remodelling properties and could be used to accelerate wound repair during the stages of scar formation when antibacterial properties are no longer required. Furthermore, it exhibits a more manifested direct bactericidal activity against *S. aureus* than Resveratrol and Dihydroquercetin, which is confirmed by the experiments in vitro [10] and in vivo. On the contrary, Dihydroquercetin displayed microbicidal properties against both *P. aeruginosa* and *C. albicans in vivo* rather than immunostimulating ones. It could be applied during the early stages of a trauma treatment in combination with antibiotics to prevent suppurations.

## 4. Materials and Methods 

### 4.1. Plant Material and Extract Procedure

The bark of Norway spruce (*P. abies* L.) was collected from debarking spruce logs from Vladimir forest (Vladimir Oblast, Russia). In the laboratory, the raw material was separated from wood residues and lichens, washed, and then air-dried at an ambient temperature for 5 days. Bark was milled and sieved to select particles between 0.5 and 0.25 mm. The bark samples for extracting polyphenols were chopped into wood-pulp (participle size was about 0.1 mm) and dried at a temperature of 40 °C. Then, we followed the method described by Ghitescu et al. for the extraction [21].

### 4.2. Separation and Identification of Polyphenols

For the HPLC separation and identification, the method by Sultana et al. was followed [22]. The identification of polyphenols (Resveratrol, Dihydroquercetin, and Dihydromyricetin) was carried out by comparing their retention times with those of authentic standards. Resveratrol and Dihydromyricetin were purchased from TransMIT Geselschaft fur Technologietrasfer mbH (Gießen, Germany). Dihydroquercetin was purchased from Favorski Irkutsk Institute of Organic Chemistry (Irkutsk, Russia). The authenticity and purity of the substances was confirmed by 1H nuclear magnetic resonance spectra made using Bruker AM-300 spectrometer (Bruker Daltonics GmbH, Brema, Germany).

The substances were dissolved in 1% sterile apyrogenic physiological saline (Escom, Moscow, Russia), sterilized using Sterile Minisart^®^ Syringe Filter, Polyethersulfone (PES), Pore Size 0.1 µm (Sartorius, Goettingen, Germany) and stored in 50 mL polystyrene tubes with a screw cap at 4 ℃ for up to one month in the dark.

Levomecol (NizhPharm JSC Nizhny Novgorod, Russia) is an ointment containing Dioxomethyltetrahydropyrimidinum (4.0%) and Chloramphenicolum (0.75%) as active agents. It is recommended for external use for treating purulent wounds, trophic ulcers, purulent and inflammatory skin diseases, boils, and burns of 2–3 degrees.

Clotrimazole (Glaxo-Wellcome, Poznan, Poland) 1% is recommended as an ointment for external use for treating infectious vaginosis caused by either *C. albicans* or other fungal species (*Malassezia furfur* (phylum Basidiomycota), *Cryptococcus neoformans, Coccidioides immitis, Trichophyton, Blastomyces dermatitidis, Sporothrix schenckii*, Epidermophyton, Microsporum, Aspergillus), or some Gram-positive bacterial pathogens, including *S. aureus*.

### 4.3. Animal Experiments

The experiments in vivo were organised according to the European Convention about the defence of vertebrates used for experiments or for any other scientific aims (Strasbourg, Mar. 18, 1986) of ETS N123 in vivarium of Kursk State Medical University. The plan for the experiment was considered by the Ethics Committee established by order of Rector of Kursk State Medical University #120 (May 12, 2016,) and approved at the session of the Committee under supervision of the chairman professor S.V. Povetkin (protocol #3, Oct 30, 2017).

Adult male Wistar rats weighing 180 ± 20.0 g and aged 3–4 months were maintained in animal rooms at 22 ℃ in a 12-h light/dark cycle and received food and water ad libitum. After the quarantine, they were placed in individual cages. The animals were randomly divided into four groups (50 animals each): (1) no infection, or infected with: (2) *S. aureus*, (3) *P. aeruginosa*, and (4) *C. albicans.* Each group was divided into five subgroups treated with (1) placebo (mock treatment with normal saline), (2) positive control (treatment with a respective reference drug), (3) experimental–Resveratrol, (4) experimental—Dihydroquercetin, and (5) experimental—Dihydromyricetin. Each subgroup was randomly divided into three echelons withdrawn from the experiment on the 3rd, 10th, and 14th day after the beginning of treatment.

### 4.4. Bacteria

The bacterial pathogens of *S. aureus* (ATCC 25923) and *P. aeruginosa* (ATCC 27853) isolated from clinical specimens were purchased from the type strain collection from Tarasevich Science State Scientific Research Institute for Standardisation and Control of Medical Biological Preparations (Moscow, Russia). The strains grew for 18–20 h. at slant IPA (meat-peptone nutrient agar) supplemented with 0.1% glucose. The fresh cultures were rinsed out with sterile normal saline, thoroughly suspended, adjusted to a concentration ~10^9^ CFU per mL using an optical turbidity standard of CCA 42-28-29-85, and used for the inoculation of wounds.

### 4.5. Fungi

The human fungal pathogen of *C. albicans* (NCTC 2625) isolated from human clinical specimens was purchased from the type strain collection from Tarasevich Science State Scientific Research Institute for Standardisation and Control of Medical Biological Preparations (Moscow, Russia). The *C. albicans* yeast was obtained using cultivation at slant meat-peptone nutrient agar supplemented with 1% glucose at 37 ℃ for 18–20 h. The cells were washed from the slant agar with sterile normal saline, thoroughly suspended, adjusted to a concentration of about 10^9^ CFU per mL using an optical turbidity standard of CCA 42-28-29-85, and used for the inoculation of wounds in rats.

### 4.6. Surgical Manipulations, Treatment, and Planimetry Assay of the Wounds

Purulent infection was modelled on rats using the method described previously by Khanin et al. [23]. The animals were anaesthetised with ether. A square 2 × 2 cm skin area on the animal’s back was thoroughly shaved, treated with a disinfectant (70% ethanol), and then the dermis and epidermis were surgically removed. Next, 1 mL of bacterial suspension containing either 10^9^ CFU/mL *S. aureus* ATCC 25923 or 10^9^ CFU/mL *S. aureus* ATCC 25923 and 10^9^ CFU/mL *P. aeruginosa* ATCC 27853 was distributed on the wound surface. To standardise wound healing conditions, the wound cavity was covered with a gauze bandage.

Thirty-six hours after wounding and infecting, all of the animals exhibited evident symptoms of suppuration and inflammation. At this point, the stitches and bandage were removed, and the wound cavity was thoroughly washed to remove pus. The wound area was measured using a sterile transparent film. Then, to treat the wounds, a 3% hydrogen peroxide specific treatment was used. The described wound treatment was repeated daily for 14 days after the beginning of the treatment.

The wounds in the control subgroup were treated with both 3% hydrogen peroxide and sterile normal saline. In the other subgroups, instead of normal saline, the wounds were treated with the compounds studied or reference drugs described in Section 2.

The animals were examined daily, and each stage of the wound healing (inflammation, granulation, and maturation (marginal epithelialisation)) was documented.

The planimetric analysis of the wound recovery percentage was performed on the 3rd, 10th, and 14th day after the start of curing. Then, one echelon (three or four animals from each subgroup) was withdrawn from the experiment on days 3 (three animals), 10 (three animals), and 14 (four animals). These animals were sacrificed using an overdose of ether anaesthesia.

The wounds were measured on different days as described in a study by Takzaree et al., using a transparent sheet, which was scanned at a resolution of 200 pcs/inch [24]. The image was acquired in Adobe Photoshop CS5 Extended format. The object was selected, and its square was calculated using a menu command “Analysis”. The average mean and a standard error (M ± Std.Err.) were calculated. The recovery percentage was calculated with the following formula:

R = S_o_ – S_x_/S_o_ × 100 (1)

where R—recovery percentage, So—wound surface on the day zero of curing, and Sx—wound surface on day X, the day of wound surface measurement.

### 4.7. Bacteriological Analysis of Microbial Load in Wound Cavity Tissue

Here, 0.1–0.5 g tissue (fibrous mass, infiltrate, and underlying derma) was sampled from a sacrificed animal under aseptic conditions, weighed (accuracy up to 0.1 mg), placed in a sterile porcelain mortar, mixed with sterile normal saline in a ratio of 1:10, and homogenised with a sterile pestle for 3 min. The homogenate was diluted 1000 times with sterile normal saline (three consequent steps of dilution 10 times using 1 mL samples), and a 100 μL aliquot of each dilution was inoculated on Petri dishes with IPA (meat—peptone nutrient agar) containing 0.1% glucose. The inoculated dishes were incubated at 37 ± 1 ℃ for 20 h and then for another day at room temperature. The colonies on each Petri dish were counted and the number of CFU was calculated per 1 g of tissue. The count was considered valid if the number of colonies varied from 30 to about 300.

### 4.8. Histological Analysis

Once the treatment was complete, the animals were sacrificed and tissue samples were prepared as described previously [24]. The biological material obtained was fixed in 10% neutral formalin. Then, the tissue samples were dissected into 1 × 1 cm fragments, washed, dehydrated, and paraffinised using standard methods. Next, 5–7 μm-thick microtome sections were stained with haematoxylin (FSR 2012/14184, ABRIS-plus R&D company, Saint Petersburg, Russia) and eosin (E-012/1000, Labiko+ LLC, Russia) ready-to-use solutions. A light microscope Leica CME (magnifications ×40, ×100, ×200, and ×400; a camera of DCM – 510) was used. Ten sections from each wound were examined and assayed. Image tools 3 software (UTHSCSA ImageTool, USA) was used to accumulate and analyse the images. The cell content close to the wound edge and underlying tissue was examined in each micrograph. The cells were categorised into polymorphonuclear and immature neutrophils, macrophages, monocytes, mast cells, fibroblasts, fibrocytes, and capillary endotheliocytes according to their morphological features (shape of the cell and the nucleus, cytoplasm/nucleus ratio) as previously described in a study by Kostiuchenok et al. [25]. The regularity of the collagen fibre alignment was assessed. The percentage of various cell types in a cell population was calculated by counting 100 cells in several non-overlapping visual fields (at least 10). The number of each cell type was shown as a percentage.

### 4.9. Statistical Analysis

Statistical analysis of research results was performed by Microsoft Excel 2007 and program "Statistics" 8.0 StatSoft. The averages of quantitative indexes and standard errors of the mean were calculated. Authenticity of distinctions of averages between the series of comparison and other series was estimated by the Mann–Whitney U test (*p* < 0.05).

## 5. Conclusions

The antimicrobial efficacy of Resveratrol and some other polyphenols is provided by the combination of both direct antibacterial and antifungal action and immunostimulating effect inside the wound. Our data indicate the ability of Resveratrol to limit the infiltration of mast cells to the wounded area, whereas the infiltration of lymphocytes and macrophages is stimulated. The infiltration of neutrophils depends on the type of pathogen. In this way, one can suppose that Resveratrol is capable of suppressing an acute antigen-dependent reaction (Th1-type response) in favour of Th2 response. This could reduce the whole wound recovery due to the synergism of antigen-dependent and antigen-independent mechanisms. Resveratrol stimulates the degradation of fibrin clots, remodelling of the collagen matrix, and vascularisation of the wound defect under recovery. Its application decreases scar formation and stimulates the restoration of hair bulbs. It is likely that this effect is partially due to the increased infiltration of macrophages which are responsible for such effects.

## 6. Patents

A patent application “Using polyphenol compounds of plant origin as a medicine for therapy of the infected wounds” was submitted to the Russian Patent Agency (registration No 2019139787, priority since 05.12.2019).

## Figures and Tables

**Table 1:** The dynamics of clinical pattern of the wound healing in the animals with uninfected wounds (M ± m).

Subgroup	Clinical Presentation			
	Disappearance of Perifocal Oedema, Day	Full Wound Cleansing, Day	Appearance of Granulation, Day	Start of Edge Epithelialisation, Day
Placebo	7.25 ± 0.25	8.25 ± 0.25	9.00 ± 0.00	9.50 ± 0.29
Resveratrol	5.25 ± 0.25 *	6.25 ± 0.25 *	6.75 ± 0.25 *	7.50 ± 0.29 *
Dihydroquercetin	5.50 ± 0.29 *	6.75 ± 0.25 *	7.00 ± 0.00 *	7.50 ± 0.29 *
Dihydromyricetin	5.50 ± 0.29 *	6.50 ± 0.29 *	7.25 ± 0.25 *	7.75 ± 0.25 *

*—*p* < 0.05 if compared to the placebo group.

**Table 2:** Dynamics of wound size changes in the animals with uninfected wounds.

Subgroups	Parameter	Day 3	Day 10	Day 14
		n = 10	n = 7	n = 4
Placebo	Wound square (cm^2^)	4.26 ± 0.38	3.21 ± 0.36	2.09 ± 0.08
	Wound reduction (%)		24.67	51.04
	Residual wound square (%)		65.58	40.49
Resveratrol	Wound square (cm^2^)	3.61 ± 0.46	1.79 ± 0.27 *	0.88 ± 0.17 *
	Wound reduction (%)		50.54	75.61
	Residual wound square (%)		41.07	17.70
Dihydroquercetin	Wound square (cm^2^)	4.62 ± 0.28	2.32 ± 0.37	0.97 ± 0.3
	Wound reduction (%)		49.77	79.00
	Residual wound square (%)		47.51	19.82
Dihydromyricetin	Wound square (cm^2^)	3.02 ± 0.29 *	1.5 ± 0.25 *	0.76 ± 0.14 *
	Wound reduction (%)		50.31	75.03
	Residual wound square (%)		49.17	23.08

*— *p* < 0.05 if being compared to the placebo group.

**Table 3:** Rate of wound healing in the experimental animals with uninfected wounds (М ± m).

Subgroup	Rate of Healing (%/day)	
	Day 10	Day 14
	n = 7	n = 4
Placebo	3.52	6.59
Resveratrol	7.22	6.27
Dihydroquercetin	7.11	7.31
Dihydromyricetin	7.19	6.18

**Table 4:** Dynamics of clinical pattern of the wound healing in the animals with the wounds infected with *S. aureus* 592 (M ± m).

Subgroup	Clinical Presentation			
	Disappearance of Perifocal Oedema, Days	Full Wound Cleansing, Days	Appearance of Granulation, Days	Start of edge Epithelialisation, Days
Placebo	9.29 ± 0.18	10.29 ± 0.18	10.43 ± 0.20	10.86 ± 0.14
Control	7.00 ± 0.32 *	8.20 ± 0.20 *	8.20 ± 0.20 *	8.20 ± 0.20 *
Resveratrol	7.43 ± 0.20 *	8.14 ± 0.14 *	8.14 ± 0.14 *	8.14 ± 0.14 *
Dihydroquercetin	8.00 ± 0.00 *^,#^	8.60 ± 0.24 *	8.20 ± 0.20 *	8.20 ± 0.20 *
Dihydromyricetin	7.29 ± 0.18 *	8.57 ± 0.2 *	8.14 ± 0.14 *	8.14 ± 0.14 *

*—*p* < 0.05 if being compared to the placebo group, ^#^—*p* < 0.05 if being compared to the control group.

**Table 5:** Dynamics of wound size changes in the animals with the wounds infected with *S. aureus* 592 (M ± m).

Subgroups	Parameter	Day 3	Day 10	Day 14
		n = 10	n = 7	n = 4
Placebo	Wound square (cm^2^)	5.53 ± 0.33	3.97 ± 0.48	2.83 ± 0.51
	Wound reduction (%)		28.22	48.86
	Residual wound square (%)		70.16	49.36
Control	Wound square (cm^2^)	4.94 ± 0.52	3.03 ± 0.19	2.7 ± 0.08
	Wound reduction (%)		38.53	45.30
	Residual wound square (%)		59.76	37.05
Resveratrol	Wound square (cm^2^)	4.09 ± 0.37 *	2.66 ± 0.41	1.46 ± 0.2 *^,#^
	Wound reducing (%)		35.05	64.25
	Residual wound square (%)		57.80	34.55
Dihydroquercetin	Wound square (cm^2^)	5.8 ± 0.38	3.39 ± 0.36	2.26 ± 0.04^#^
	Wound reduction (%)		41.55	61.09
	Residual wound square (%)		57.68	39.25
Dihydromyricetin	Wound square (cm^2^)	5.15 ± 0.53	2.77 ± 0.3	1.71 ± 0.28
	Wound reduction (%)		46.34	66.87
	Residual wound square (%)		58.40	36.86

*—*p* < 0.05 if being compared to the placebo group, ^#^—*p* < 0.05 if being compared to the control group.

**Table 6:** Rate of wound healing in the experimental animals with the wound infected with *S. aureus* 592 (М ± m).

Subgroups	Rate of Healing (%/day)	
	10 Days	14 Days
	n = 7	n = 4
Placebo	4.03	5.16
Control	5.50	1.69
Resveratrol	5.01	7.30
Dihydroquercetin	5.94	4.89
Dihydromyricetin	6.62	5.13

**Table 7:** Dynamics of bacterial load in the wounds infected with *S. aureus* 592 (M ± m).

Animal	Day 3				
	Placebo	Control	Resveratrol	Dihydroquercetin	Dihydromyricetin
**1**	600.2 × 10^4^	1 × 10^4^	100.3 × 10^4^	3.6 × 10^4^	3.6 × 10^4^
**2**	8.5 × 10^4^	19.1 × 10^4^	10.8 × 10^4^	550.4 × 10^4^	2.2 × 10^4^
**3**	26.3 × 10^4^	750.3 × 10^4^	5.3 × 10^4^	17.6 × 10^4^	39.1 × 10^4^
**Day 10**					
**1**	100.4 × 10^4^	28.3 × 10^4^	7.4 × 10^4^	10.8 × 10^4^	16.9 × 10^4^
**2**	24.5 × 10^4^	1.3 × 10^4^	58.1 × 10^4^	180.3 × 10^4^	11.2 × 10^4^
**3**	24.7 × 10^4^	1.2 × 10^4^	40.8 × 10^4^	56.9 × 10^4^	9.4 × 10^4^
**Day 14**					
**1**	82.2 × 10^4^	25.1 × 10^4^	9.9 × 10^4^	2.1 × 10^4^	46.2 × 10^4^
**2**	12.6 × 10^4^	99.1 × 10^4^	11.2 × 10^4^	13.6 × 10^4^	63.2 × 10^4^
**3**	-	-	13.5 × 10^4^	-	39.4 × 10^4^

**Table 8:** Dynamics of clinical pattern of the wound healing in the animals with the wounds infected with *P. aeruginosa* (M ± m).

Subgroups	Clinical Presentation			
	Disappearance of Perifocal Oedema, Days	Disappearance of Perifocal Oedema, Days	Disappearance of Perifocal Oedema, Days	Disappearance of Perifocal Oedema, Days
Placebo	9.17 ± 0.17	10.33 ± 0.21	10.50 ± 0.22	11.0 ± 0.00
Control	7.00 ± 0.00 *	8.00 ± 0.00 *	8.00 ± 00 *	8.00 ± 0.00 *
Resveratrol	7.14 ± 0.14 *	8.14 ± 0.14 *	8.14 ± 0.14 *	8.14 ± 0.14 *
Dihydroquercetin	7.14 ± 0.14 *	8.14 ± 0.14 *	8.14 ± 0.14 *	8.14 ± 0.14 *
Dihydromyricetin	7.20 ± 0.20 *	8.20 ± 0.20 *	8.20 ± 0.20 *	8.20 ± 0.20 *

*— *p* < 0.05 if being compared to the placebo group, ^#^— *p* < 0.05 if being compared to the control group.

**Table 9:** Dynamics of wound size changes in the animals with the wounds infected with *P. aeruginosa* (М ± m).

Subgroups	Parameter	Day 3	Day 10	Day 14
		n = 10	n = 7	n = 4
Placebo	Wound square (cm^2^)	6.33 ± 0.7	5.09 ± 0.44	2.63 ± 0.2
	Wound reduction (%)		19.56	58.40
	Residual wound square (%)		72.67	46.42
Control	Wound square (cm^2^)	4.86 ± 0.49	2.84 ± 0.46 *	1.17 ± 0.06 *
	Wound reduction (%)		41.49	75.91
	Residual wound square (%)		54.35	32.99
Resveratrol	Wound square (cm^2^)	5.61 ± 0.38	3.72 ± 0.7	1.41 ± 0.4
	Wound reduction (%)		33.59	74.93
	Residual wound square (%)		60.80	28.03
Dihydroquercetin	Wound square (cm^2^)	6.73 ± 0.45	4.10 ± 0.24 ^#^	1.86 ± 0.37 ^#^
	Wound reduction (%)		39.13	72.44
	Residual wound square (%)		65.84	36.33
Dihydromyricetin	Wound square (cm^2^)	5.81 ± 0.45	3.37 ± 0.38 *	1.63 ± 0.3
	Wound reduction (%)		42.06	72.03
	Residual wound square (%)		63.17	28.95

*—*p* < 0.05 if being compared to the placebo group, ^#^—*p* < 0.05 if being compared to the control group.

**Table 10:** Rate of wound healing in the experimental animals with the wound infected with *P. aeruginosa* (М±m).

Subgroups	Rate of Healing (%/day)	
	10 Days	14 Days
	n = 7	n = 4
Placebo	2.79	9.71
Control	5.93	8.61
Resveratrol	4.80	10.34
Dihydroquercetin	5.59	8.33
Dihydromyricetin	6.01	7.49

**Table 11:** Dynamics of bacterial load in the wounds infected with *P. aeruginosa* (M ± m).

Animal	Day 3				
	Placebo	Control	Resveratrol	Dihydroquercetin	Dihydromyricetin
**1**	5.8 × 10^4^	0	84.5 × 10^4^	0	68.4 × 10^4^
**2**	91.4 × 10^4^	14.7 × 10^4^	20.6 × 10^4^	31.3 × 10^4^	0
**3**	1.2 × 10^4^	6.7 × 10^4^	1.9 × 10^4^	0	23.5 × 10^4^
**Day 10**					
**1**	25.9 × 10^4^	1.0 × 10^4^	149.4 × 10^4^	28.0 × 10^4^	37.0 × 10^4^
**2**	111.4 × 10^4^	18.1 × 10^4^	72.4 × 10^4^	117.2 × 10^4^	92.5 × 10^4^
**3**	0	56.5 × 10^4^	62.7 × 10^4^	3.6 × 10^4^	100.7 × 10^4^
**Day 14**					
**1**	93.0 × 10^4^	3.4 × 10^4^	17.2 × 10^4^	400.0 × 10^4^	1.5 × 10^4^
**2**	16.4 × 10^4^	0	-	740.0 × 10^4^	19.5 × 10^4^
**3**	34.6 × 10^4^	-	-	16.6 × 10^4^	-

**Table 12:** Dynamics of clinical pattern of the wound healing in the animals with the wounds infected with *C. albicans* (M ± m).

Subgroup	Clinical presentation			
	Disappearance of Perifocal Oedema, Days	Disappearance of Perifocal Oedema, Days	Disappearance of Perifocal Oedema, Days	Disappearance of Perifocal Oedema, Days
Placebo	9.4 ± 0.24	10.4 ± 0.24	10.6 ± 0.24	11.2 ± 0.2
Control	6.71 ± 0.18 *	7.71 ± 0.18 *	7.71 ± 0.18 *	7.86 ± 0.26 *
Resveratrol	7 ± 0 *	8 ± 0 *	8 ± 0 *	8.14 ± 0.14 *
Dihydroquercetin	5.29 ± 0.18 *^,#^	8.29 ± 0.18 *^,#^	8.43 ± 0.2 *^,#^	8.57 ± 0.2 *
Dihydromyricetin	7.14 ± 0.14 *	8.14 ± 0.14 *	8.29 ± 0.18 *^,#^	8.43 ± 0.2 *

*—*p* < 0.05 if being compared to the placebo group, ^#^—*p* < 0.05 if being compared to the control group.

**Table 13:** Dynamics of wound size changes in the animals with the wounds infected with *C. albicans* (M ± m).

Subgroup	Parameter	Day 3	Day 10	Day 14
		n = 10	n = 7	n = 4
Placebo	Wound square (cm^2^)	6.99 ± 0.52	5.41 ± 0.62	4.08 ± 0.44
	Wound reduction (%)		22.61	41.61
	Residual wound square (%)		78.43	55.98
Control	Wound square (cm^2^)	6.21 ± 0.68	4.00 ± 0.57	3.09 ± 0.7
	Wound reduction (%)		35.53	50.22
	Residual wound square (%)		58.17	37.35
Resveratrol	Wound square (cm^2^)	5.70 ± 0.63	2.87 ± 0.44 *	0.93 ± 0.26 *^,#^
	Wound reduction (%)		49.73	83.65
	Residual wound square (%)		55.69	20.69
Dihydroquercetin	Wound square (cm^2^)	4.59 ± 0.43 *	3.28 ± 0.45 *	2.19 ± 0.37 *
	Wound reduction (%)		28.48	52.27
	Residual wound square (%)		66.30	42.15
Dihydromyricetin	Wound square (cm^2^)	5.79 ± 0.31	3.32 ± 0.32 *	2.41 ± 0.57
	Wound reduction (%)		42.62	58.38
	Residual wound square (%)		59.61	41.67

*—*p* < 0.05 if being compared to the placebo group, ^#^—*p* < 0.05 if being compared to the control group.

**Table 14:** The rate of wound healing in the experimental animals with the wound infected with *C. albicans* (М ± m).

Subgroup	Rate of Healing (%/day)	
	10 Days	14 Days
	n = 7	n = 4
Placebo	3.23	4.75
Control	5.08	3.67
Resveratrol	7.10	8.48
Dihydroquercetin	4.07	5.95
Dihydromyricetin	6.09	3.94

**Table 15:** Dsynamics of yeast load in the wounds infected with *C. albicans* (M ± m).

Animal	Day 3				
	Placebo	Control	Resveratrol	Dihydroquercetin	Dihydromyricetin
**1**	0	10.3 × 10^4^	0	0	0
**2**	0	0	0	1.6 × 10^4^	0
**3**	4.9 × 10^4^	0	0	0	3.6 × 10^4^
**Day 10**					
**1**	0.5 × 10^4^	3.2 × 10^4^	1.5 × 10^4^	0	12 × 10^4^
**2**	0	1.7 × 10^4^	0.8 × 10^4^	0	2.5 × 10^4^
**3**	30.6 × 10^4^	0	0	1.6 × 10^4^	18.4 × 10^4^
**Day 14**					
**1**	0	0	0	0	0
**2**	0	0	0	0	0
**3**	0	0	0	0	0
